# Vocal fold injury models in rats: a literature review on techniques and methodology

**DOI:** 10.25122/jml-2022-0032

**Published:** 2022-03

**Authors:** Peter Laszlo Ujvary, Cristina Maria Blebea, Alma Aurelia Maniu, Sever Pop, Orsolya Sarpataki, Marcel Cosgarea

**Affiliations:** 1. Department of Otolaryngology, Iuliu Hatieganu University of Medicine and Pharmacy, Cluj-Napoca, Romania; 2. Department of Physiology, University of Agricultural Sciences and Veterinary Medicine, Cluj-Napoca, Romania

**Keywords:** rat, vocal fold, review, injury, methodology

## Abstract

This study reviewed the current literature on technical aspects regarding controlled vocal fold injuries in the rat model. Data from PubMed, Embase, and Scopus database for English language literature was collected to identify methodological steps leading to a controlled surgical injury of the rat vocal fold. Inclusion criteria: full disclosure of anesthesia protocol, positioning of the rat for surgery, vocal fold visualization method, instrumentation for vocal fold injury, vocal fold injury type. Articles with partial contribution were evaluated and separately included due to the limited number of original methodologies. 724 articles were screened, and eleven articles were included in the analysis. Anesthesia: ketamine hydrochloride and xylazine hydrochloride varied in dose from 45 mg/kg and 4.5 mg/kg to 100 mg/kg and 10 mg/kg. Visualization: The preferred method was the 1.9 mm, 25–30 degree endoscopes. The widest diameter endoscope used was 2.7 mm with a 0 or 30 degree angle of view. Instruments for lesion induction range from 18 to 31G needles, microscissors, micro forceps to potassium titanyl phosphate, and blue light lasers. Injury types: vocal fold stripping was the main injury type, followed by vocal fold scarring and charring. One article describes scaffold implantation with injury to the superior aspect of the vocal fold. Rats are good candidates for in vivo larynx and vocal folds research. A more standardized approach should be considered regarding the type of vocal fold injury to ease data comparison.

## INTRODUCTION

Voice quality after vocal fold injury, scarring, and subsequent surgery is still a significant concern for the clinician and patient. The vocal folds are uniquely layered structures that consist of stratified non-keratinized squamous epithelium, lamina propria (LP), and the thyroarytenoid muscles [1]. Primarily, the vibratory property of the vocal folds depends on the size and the extracellular matrix (EM) constituents of the LP [2–3]. Exposing the LP due to epithelial injury alters the composition of collagen, elastin, and glycosaminoglycan [4] in the EM and impairs vibratory function. These changes will clinically manifest as dysphonia [5].

**Figure 1.**
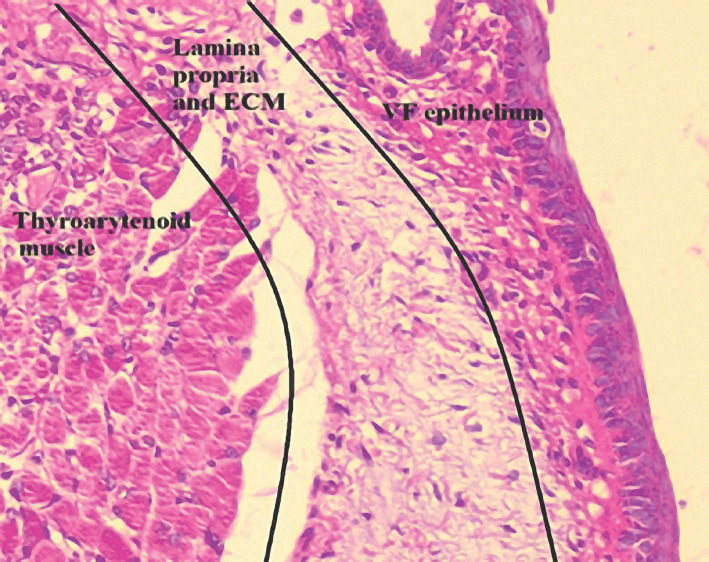
Histological layers of the normal rat vocal fold. The three layers of the rat vocal fold; 200x magnification with HE staining. VF – vocal fold; ECM – extra cellular matrix.

**Figure 2.**
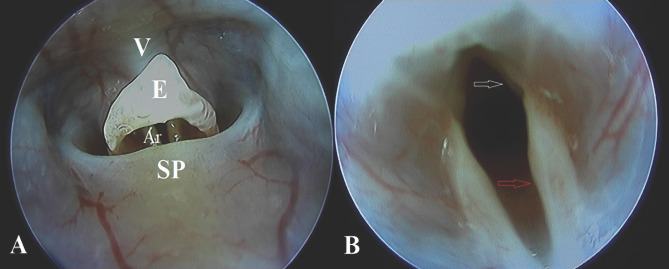
Macroscopic anatomy of the rat vocal fold and surrounding region. A: V – vallecular; E – epiglottis; SP – soft palate; Ar – Left arytenoid cartilage. B: The right membranous the vocal is marked with a white arrow and can be found inferior to the base of the epiglottis. The arytenoid is marked with the red arrow. These are important landmarks helping to identify the true membranous vocal folds.

Animal models have been widely used to document healing, scaring, and biomechanics of the vocal folds. The rat model is the most widely used, partially due to the same tri-layered vocal fold structure as humans and presents similar fibrous protein composition of the EM. Also, a great amount of research data is already available regarding chronic scarring of the vocal fold compared to the canine of the rabbit model [6–7]. A rat vocal fold injury model is difficult to replicate even throughout the study groups due to the lack of standardization and reproducibility of the vocal fold lesions themselves. Imaizumi *et al.* emphasized the importance of depth and length of a controlled injury in experimental studies by cross-referencing rat vocal fold injuries to human vocal fold pathology and excision extent [8]. Understanding vocal fold anatomy, histological layer succession, and the space provided by the rat pharyngeal lumen (Figure 1 and Figure 2) are key in performing controlled in vivo laryngeal surgery in concordance with the scope of the study. Although the rat model holds several advantages over other species, technical difficulty may arise when choosing the appropriate surgical methodology for the scope of the study.

This review article focuses on technical aspects of controlled vocal fold injury in rat models to synthesize and improve current knowledge on methodology and assist further experimental research regarding in vivo vocal fold healing in the rat model.

## MATERIAL AND METHODS

A literature review was conducted in March 2021, following the Preferred Reporting Items for Systematic Reviews and Meta-Analysis (PRISMA) guidelines.

### Literature search criteria

A primary electronic MEDLINE (PubMed), Embase, and Scopus database search for English language literature were carried out with advanced search criteria using MeSH terms: "rat" and "vocal fold". No publication period restriction was used. The scope was to identify all possible articles in these databases, discussing surgical injury to the rat vocal fold, regardless of the scope of the study.

### Selection criteria

The objectives were to systematically identify the methodological steps that lead to a controlled surgical injury of the rat vocal fold. Based on the title and abstract, all identified articles were independently screened by two authors for inclusion. Articles that did not indicate a vocal fold injury or healing process were excluded. The included articles were screened as full-text versions.

We established five criteria as a part of a controlled vocal fold injury workflow for a methodology to be eligible. Criteria were as follows: full disclosure of anesthesia protocol; positioning of the rat for surgery; vocal fold visualization method; instrumentation for vocal fold injury; mentioning the type of vocal fold lesion created.

 First, articles from the same author were screened to determine if the same methodological steps apply. If the methodology of the surgical injury was the same, the methodology discussed in the most recent article was excluded. Studies that cited and integrally used methods already published in the literature were eliminated. We also excluded the referenced article if the methodology for the vocal fold injury model was also cited from a previous article. The process was repeated until the original methodology was found. Furthermore, all articles discussing chronic vocal fold injury; laryngeal nerve injury, including other experimental animals of which the title and abstract screening did not pick up, were also excluded.

After including only the articles that discuss acute vocal fold injury in a rat model, a second screening was applied to include articles that mention all five methodological steps discussed above.

Finally, all original articles that met inclusion criteria were noted in an Excel spreadsheet and qualitatively analyzed. If discrepancies arose regarding any point of the screening process, the senior author stepped in as arbitrator.

Given the limited number of original methodologies found, a secondary manual search was conducted to include articles with a partial contribution. The partial contribution was defined as articles not mentioning all steps of the methodological process to be eligible for inclusion but adding value through original insight to the vocal fold injury process.

## RESULTS

### Study characteristics

**Figure 3.**
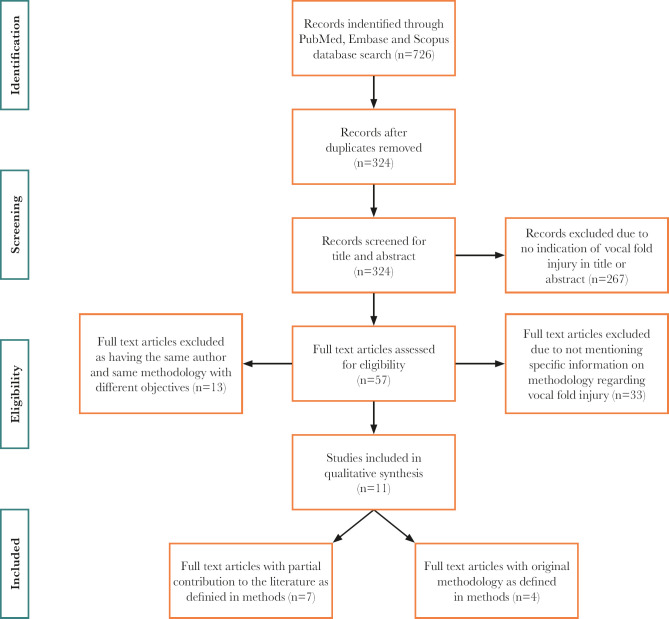
PRISMA (Preferred Reporting Items for Systematic Reviews and Meta-analyses) flowchart summarizing the search results and the application of eligibility criteria.

The database search resulted in a total of 726 publications. MEDLINE n=266; Embase n=214; Scopus n=246. After removing duplicates, 326 articles were included for analysis. The selection process is illustrated in Figure 3. Fifty-seven articles were selected for full-text analysis, eleven of which were included for qualitative analysis. Seven articles that did not meet all inclusion criteria but partially contributed insight into the surgical methodological steps are synthesized in Table 1. Four articles that met all inclusion criteria as original methodologies are synthesized in Table 2.

**Table 1.** The list of methodologies with partial contribution.

Nr.	Author	Year	Visualization	Lesion induction	Positioning of animal	Type of lesion	Anesthesia
**1.**	Lin R. J *et al.* [9]	2020	2.5 mm 0 degrees endoscope	445 nm BL LASER at 2 W, 10 ms through nr. 5 Frasier cannula	Semi vertical position on custom operating platform	Superficial charring of the vocal fold	Not mentioned
**2.**	Kim C-S *et al.* [10]	2018	2.7 mm rigid endoscope	Micro scissors	Semi vertical position on a custom made platform	Vocal fold stripping	Induction with isoflurane IP inj. of K: 100 mg/kg X: 10 mg/kg
**3.**	T. Morisaki *et al.* [11]	2018	2.7 mm 30 degrees endoscope	25 G needle	Semi vertical position on a custom made platform	Vocal fold stripping	IP inj. of K: 45 mg/kg X: 4.5mg/kg
**4.**	R. Suzuki *et al.* [12]	2017	30 degree rigid endoscope	Micro scissors and microforceps	Semi vertical position on a custom made platform	Vocal fold stripping	IP inj. of K: 45mg/kg X: 4.5mg/kg
**5.**	M. Gugatschka *et al.* [13]	2011	Endoscope – no details mentioned	90 degree bent scissor	Vertical position on custom made platform	Vocal fold scarring	Induction with diethyl ether IP inj. of K: 45 mg/kg X: 4.5 mg/kg Topical anesthesia (5% lidocaine)
**6.**	B. H. Q. Johnson *et al.* [14]	2010	1.9 mm diameter 30 degree endoscope	27 gauge needle	Semi vertical position on custom made platform	Scarring of mid-membranous portion of the vocal folds.	Induction with isoflourane IP inj. of K: 90 mg/kg X: 9 mg/kg; IP inj of atropine 40 ug/kg
**7.**	Ohno *et al.* [15]	2009	1.9 mm 30 degree endoscope	22 gauge needle	Custom-made operating platform	Vocal fold stripping	IM inj. of K: 60 mg/kg X: 6 mg/kg

BL – blue light; K – Ketamine; X – Xilazine; IP – intraperitoneal; IM – intramuscular.

**Table 2.** List of original methodologies considering all inclusion criteria.

Nr.	Author	Year	Visualization	Lesion induction	Positioning of animal	Type of lesion	Anesthesia
**1.**	Mallur P.S *et al.* [16]	2009	2.5 mm 0 degrees endoscope; retraction on tongue; spring wire laryngoscope; retraction of the epiglottis with a blunt probe	532 nm KTP LASER at 10 W 20 ms through a 0.4 mm fiber through size 5 Frasier suction tip	Supine in a horizontal position	Blanching of the vocal fold mucosa	IP inj. of K: 80 mg/kg X: 8 mg/kg
**2.**	Xu. C *et al.* [17]	2009	Surgical microscope and 4 mm ear speculum	A custom tool from an 18 G spinal needle	Custom made platform in semi-vertical supine position	0.8 mm incision on the superior surface of the posterior one-third of the vocal fold of each vocal fold	Induction by inhalation of 4% isoflurane; K: 65 mg/kg X: 6.5 mg/kg Carprofen 5 mg/kg 100 µL 2% lidocaine on vocal folds
**3.**	Tateya T *et al.* [18]	2005	1.9 mm 25 degrees endoscope; spring wire laryngoscope	25 G needle and microscissors	Semi vertical position on a custom operating platform	Vocal fold stripping	Induction with isoflourane delivered at 0.8–1.5 L/minute; IP inj. of K: 90 mg/kg X: 9 mg/kg Topical vocal fold anaesthesia with 5% lidocaine
**4.**	Kanemaru S *et al.* [19]	2005	Direct visualisation through sub-cricoid incision	32 G needle	Supine in a horizontal position	Vocal fold stripping	Induction with diethyl ether; IM inj. of K: 80 mg/kg X: 6 mg/kg

KTP – potassium titanyl phosphate; K – Ketamine; X – Xilazine; IP – intraperitoneal; IM – intramuscular.

### Anesthesia and medication

Anesthesia protocols varied in terms of dose and means of administration. Induction anesthesia was not carried out in all instances. Ketamine hydrochloride and xylazine hydrochloride were used in all cases. When induction anesthesia was preferred, isoflurane [10, 14, 17, 18] or diethyl ether [13, 19] were used. Ketamine hydrochloride and xylazine hydrochloride varied in terms of dose from 45 mg/kg and 4.5 mg/kg [11–13] to 100 mg/kg and 10 mg/kg [10]. The preferred method of drug delivery was intraperitoneal. Intramuscular delivery was only used in two cases [7, 19]. The use of Atropine sulfate 0.005 mg/kg and 5% topical lidocaine was discussed by Tateya *et al.* [18]. Carprofen 5 mg/kg was used by Xu C. *et al.* [17].

### Visualization of the rat larynx

Visualization of the rat larynx was carried out using different methods. Endoscopes were preferred over surgical microscopes or direct visualization. The 1.9 mm, 25–30 degree endoscope was the preferred method [14, 15, 18]. The widest diameter endoscope used was 2.7 mm with a 30 degree angle of view [11]. The surgical microscope was used in one case [17].

In order to secure the endo-luminal view and pharyngeal patency, a series of custom-made laryngoscopes have been described. A 4 mm ear speculum was used by inserting it through the oral cavity to act as a suspension device [17]. Another method for securing the endoluminal patency was a custom-made spring wire device laryngoscope [16, 18]. Direct visualization of the vocal fold without optical instruments was described by Kanemaru S. *et al.* [19], using a sub-cricoid incision to expose the subglottic region to visualize the vocal folds directly.

### Instruments used for acute controlled injury

The controlled vocal fold injury instrumentation was divided into cold instruments and surgical lasers. Nine authors preferred cold instruments, and two authors used either potassium titanyl phosphate (KTP) or blue light (BL) laser as a means of injury. Cold instruments ranged from micro-scissors and micro forceps to different gauge needles. The smallest needle size was 32 G [19], and the biggest diameter needle was 18 G [17]. The two original methodologies using lasers as a means of controlled injury channeled the energy through an nr. 5 Frasier suction cannula. The energy used for the KTP laser (532 nm) was at 10 W, 20 ms through a 0.4 mm fiber. For the BL laser (445 nm), the energy output was set at 2 W with 10 ms bursts.

### Injury type

Seven authors describing the use of cold instruments describe vocal fold stripping (with exposure of the thyroarytenoid muscle). Another injury type was described to facilitate the implantation of bioactive scaffolds. For this purpose, an 18 G modified spinal needle was used to create a 0.8 mm longitudinal incision at the superior surface on the posterior 1/3 of the rat vocal fold [17]. Grasping micro-forceps were used to induce focal lesion of the vocal by injuring the middle 1/3 of the vocal fold to create a model for membranous vocal fold scarring [14]. The two articles describing a thermal injury model using lasers describe charring and blanching of the vocal folds as a mean injury [9, 16].

## DISCUSSIONS

Experimental models permit the assessment of diverse bioactive agents on vocal fold wound healing that subsequently could translate into clinical application [20].

The rat model is well-established in investigating structural and functional modifications in all organ systems. Given the overall small size of rats and the 1 mm membranous vocal fold length, it is challenging to achieve consistency regarding vocal fold lesions throughout study groups [6, 21].

To the best of our knowledge, Imaizumi *et al.* [8] were the first to address the problem of consistency and comparability by describing and providing structure for the vocal fold injury model in rats. They emphasized the importance of the correlation between in vivo vocal fold injury in experimental medicine and the cordectomy classification proposed by Remacle and the European Laryngological [22]. The observations are essential for moving forward as they provide a classification for further studies, grouping vocal fold lesions in subepithelial, transmucosal, and transmuscular.

The literature regarding in-depth descriptions of the vocal fold injury process of the rat is scarce. Our review aimed to describe and synthesize various methodological steps for a controlled vocal fold injury in the rat model.

### Vocal fold injury types

#### Vocal fold stripping (transmucosal injury)

To the best of our knowledge, vocal fold stripping in the rat model was first described by Tateya *et al.* [18] and subsequently gained the most traction among researchers (Tables 1 and 2). The definition of vocal fold stripping in the rat model would correspond to a transmucosal injury described later by Imaizumi *et al.* [8]. Earlier authors mention the exposure and visualization of the thyroarytenoid muscle, which would imply removing the deeper layers of the LP and not just the epithelium and superficial layer of the LP. This type of vocal fold injury would translate to clinical practice as a type II cordectomy (subligamental cordectomy). The vocal fold stripping model is a well-established first step to address the complex nature of EMC modifications, vocal fold scarring, and fibrosis using various solutions from tissue engineering to stem cells, growth factors, and antifibrotic agents [10–12, 15, 18, 19, 23, 24]. Instrumentation tends to be similar, using small gauge needles for the procedure. Needle size ranges from 22 to 26 G with auxiliary instruments helping with manipulation (micro forceps and scissors).

#### Vocal fold scarring (subepithelial lesion) and superficial thermal injury

Superficial lesions of rat vocal fold were described using cold instruments and lasers. Cold instruments used for vocal fold scarring are twenty-seven gauge needles and 90° micro scissors. The instruments were used to scar the membranous portion of the vocal folds [13–14].

This form of injury would correspond to the subepithelial injury model [8] or, in clinical terms, to a type I cordectomy. These methods could be used to study reepithelization and modifications of the superficial layer of the LP without disturbing the vocal ligament.

Superficial epithelial blanching and charring of the vocal fold were achieved with the help of KTP and blue light (BL) laser. Mallur PS *et al.* described using the KTP on the rat vocal fold epithelium to replicate potential modifications in the clinical setting. Consistent blanching was achieved with a 10 W 20 ms pulse. Higher power led to eschar formation. No modification of the underlying cellular architecture was noted other than cellular infiltration. The energy of the 532 nm KTP laser was delivered with a 0.4 mm fiber introduced through a number 5 Frasier tip suction handpiece [16].

Compared to the KTP, the BL laser is a novel technique for laryngeal surgery. The epithelial effects and injury depth were assessed by Lin RJ *et al.* [9]. They evaluated the effects of the BL laser on the vocal fold epithelium and the LP. The energy was delivered at a 2 W and 10 ms pulse to simulate the same clinical appearance and effects of the procedure as described before (blanching and charring). They noted no significant destruction of vocal fold cellular architecture but significant postsurgical submucosal inflammation (day 1). Long-term comparison (90 days) between KTP and BL laser showed significantly higher fibrosis in the KTP group.

#### Surgical model for implantable materials in the rat vocal fold

Engineered synthetic scaffold implantation shows promise in vocal fold reconstruction and regeneration [25]. As far as we are aware, Xu. C *et al.* [17] article is the only one that gives surgical insight for scaffold implantation in the rat model. The authors describe the implantation of an acellular xenogeneic extracellular matrix scaffold through a 0.8 mm incision on the superior surface of the posterior one-third of the vocal fold. For this purpose, a custom-made 18 G spinal needle was used under the guidance of an operating microscope.

### Visualization

Endoscopes are the most common means of visualization. Endoscope size ranged from 1.9 mm to 2.7 mm. Endoscope angle was 25–30 degrees in most cases [11, 12, 14, 15, 18] and 0 degrees only in 2 cases [9, 16]. Proper visualization is essential, and the 25–30s degree, small diameter endoscopes provide the best angle and room for manipulation. Even though optimal visibility is provided, care should be taken regarding time exposure due to possible thermal injury of the surrounding tissues. It is recommended to use minimal light intensity that provides good visibility and avoid touching the mucosa. Also, limit the continuous exposure to less than 10 minutes [26, 27]. Xu C *et al.* [17] used a surgical microscope to aid scaffold implantation. This procedure differs from other types of vocal fold injuries by positioning the incision in the posterior and superior aspects of the vocal folds. In this case, the pharyngeal lumen patency was secured using an ear speculum. Other devices were mentioned to aid the visualization of the vocal folds and were described as custom-made spring wire laryngoscopes [16, 18]. These devices were fabricated from a 1 mm steel wire and were shaped to a coil at the middle, providing counter tension to the tongue base and posterior pharyngeal wall. We have learned so far that the most suitable instruments for visualizing the true vocal folds that lie deep to the epiglottis would be smaller diameter endoscopes (1.9–2.4 mm) with a 25–30 degree angle. Procedures regarding scaffold implantations, vocal fold injections, injuries to the posterior 1/3 of the vocal folds, or arytenoid surgery would best benefit from microscopic guidance. Historically, Kanemaru *et al.* were one of the first to use rats in laryngeal studies. They describe a sub-cricoid incision to visualize the true vocal folds directly and provoke injury using a 34 G needle. Nowadays, with optical devices, this technique is no longer useful. In most cases, the positioning of the rat for surgery is in posterior decubitus and a near-vertical position. A series of custom-made operating platforms have been described and illustrated [12, 13, 16, 18].

### Anesthesia and medication

There is no standardized anesthesia protocol for laryngeal surgery in rats. Ketamine and Xylazine were used with or without induction anesthesia in all instances. The ketamine dose ranged from 45 mg/kg to 100 mg/kg and the Xylazine dose ranged from 4.5 mg/kg to 10 mg/kg. We would argue that the higher dose ranges would be necessary in most cases because the vocal fold injury process itself is just the first step of the methodology in many experiments. Applying diverse bioactive agents or scaffold implantation would lengthen the time of the procedure.

Nevertheless, to the best of our knowledge, there is only one study to support the optimal anesthesia dose regarding experiments limited to the rat larynx [28]. According to Suzuki T *et al.*, a 67.5 mg/kg ketamine and a 6.75 mg/kg xylazine dose would be too low, and epiglottal movement would still be present. An optimal minimal intraperitoneal dose of 90.0 mg/kg ketamine hydrochloride and 9.0 mg/kg xylazine hydrochloride would provide sufficient anesthesia for the placement of a suspension laryngoscope.

Additional medication was prescribed in a few cases. Atropine sulfate in a dose of 0.005 mg/kg can be administered to reduce sputum secretion. A 2–5% topical lidocaine can be delivered to the vocal folds to provide additional local anesthesia [17, 18]. The use of 5 mg/kg Carprofen was discussed in one case where the probability of laryngeal edema was higher given the extent of the lesion [17].

### Clinical interest and limitations of current injury models

All articles contributed to the methodology and technique of performing vocal fold lesions in the rat model following a particular interest. The studies also give significant insight regarding cell messaging, extracellular matrix composition, and understanding of the healing cascade and the injured vocal fold scarring. Understanding the basic physiology and cell biology of healing and scarring gives the opportunity to further study bioactive agents that can serve as adjuvant treatment options in clinical practice [29]. Bioactive agents could reduce the need for prolonged voice rest or prevent vocal fold scarring after trauma or phonosurgical intervention.

Although precise and replicable vocal fold injury models (superficial epithelial injury and vocal fold stripping) have been described up to date, there is a lack in the literature regarding a subepithelial surgical model as described by Imaizumi *et al.* [8]. Only two methodologies describe this type of injury [13, 14], and the injuries provoked were only focal lesions of the vocal fold. We believe that a more elaborate subepithelial injury model would better replicate a type I cordectomy in humans, thus serving as a more practical starting point in bridging the gap between experimental vocal fold models and clinical practice. We believe that the described injury models are appropriate to analyze cellular modifications and observe scarring of the vocal fold, but a more adapted surgical model should be used to address specific problems of the superficial lamina propria.

## CONCLUSIONS

Rats are good candidates for in vivo larynx and vocal folds research. Although the small size may impede consistent vocal fold injury, a vast amount of research data is already available. We believe that a more standardized approach should be considered regarding the type of vocal fold injury. Transmucosal injuries (vocal fold stripping) are most widely used as a means of injury. A great variety of instruments can be used to achieve the desired length and depth of the lesion. Angled endoscopes permit the best visualization of the true vocal folds, but a surgical microscope can be used in cases where exposure of the posterior glottis is sufficient. Anesthesia doses vary greatly, but higher doses are required to limit the reflex movement of the epiglottis.

## ACKNOWLEDGMENTS

### Conflict of interest

The authors declare no conflict of interest.

### Personal thanks

We thank our colleague, Dr. Muresan Mihaela, for providing the histological analysis of the normal rat larynx.

### Authorship

PLU contributed to conceptualization, methodology, data collection, analysis, writing the original draft, and editing the manuscript. CMB contributed to data analysis, data collection, and writing of the original draft and manuscript. AAM contributed to editing the manuscript and writing the original draft. SP contributed to editing the manuscript and data analysis. OS contributed to editing the original draft and data curation. MC contributed to the conceptualization, methodology, and editing of the manuscript.
